# Roles and Responsibilities in Pharmacy Practice as Determinants of Burnout: A Comparative Cross-Sectional Survey of Community Pharmacists and Pharmacy Assistants in the Northeastern Region of Bulgaria

**DOI:** 10.3390/pharmacy14010026

**Published:** 2026-02-03

**Authors:** Mariya Ivanova, Antoaneta Tsvetkova, Anna Todorova

**Affiliations:** 1 Department of Organisation and Economics of Pharmacy, Faculty of Pharmacy, Medical University of Varna, 9002 Varna, Bulgaria; anna.todorova@mu-varna.bg; 2 Medical College, Medical University of Varna, 9002 Varna, Bulgaria; antoaneta.tsvetkova@mu-varna.bg

**Keywords:** burnout, community pharmacy, master pharmacists, assistant pharmacists, work environment, occupational stress

## Abstract

Background: Burnout is a significant occupational risk among healthcare professionals, including community pharmacy staff, whose differing roles and responsibilities may influence burnout determinants. This study aimed to compare burnout levels and associated work characteristics between master pharmacists (MPs) and assistant pharmacists (APs) working in community pharmacies in Northeastern Bulgaria. Methods: A cross-sectional observational survey was conducted between November 2023 and December 2024 using an anonymous, self-administered online questionnaire completed by 221 MPs and 151 APs. Burnout was assessed using the Maslach Burnout Inventory—Human Services Survey for Medical Personnel, measuring emotional exhaustion (EE), depersonalization (DP), and personal accomplishment (PA). Work characteristics were evaluated using items adapted from an internationally recognized European Commission guideline on occupational health and safety risks in the healthcare sector. Results: High levels of EE and DP were observed in both groups, with no statistically significant differences in mean burnout scores. Age and years of professional experience were not significantly associated with burnout. However, work environment factors differed: poor team communication and a negative workplace climate affected both groups, whereas lack of recognition and support was more influential for MPs, and physical workload and frequent interruptions were more prominent stressors for APs. Conclusions: Burnout is prevalent among community pharmacy professionals, with role-specific organizational factors shaping its determinants and highlighting the need for targeted preventive strategies.

## 1. Introduction

Burnout is a work-related phenomenon defined in ICD-11 as the result of chronic occupational stress that has not been successfully managed. It is characterized by three dimensions: (1) emotional exhaustion (EE), (2) depersonalization (DP), and (3) reduced professional efficacy/personal accomplishment (PA) [1]. Burnout is most commonly assessed with the validated Maslach Burnout Inventory—Human Services Survey for Medical Personnel (MBI-HSS [MP]), which encompasses these three subscales [2,3,4]. Each dimension exerts a different influence on behaviour and attitudes toward work processes: EE reflects fatigue, depletion, and lack of energy; DP encompasses negativism, detachment, and irritability towards work and people; low PA is associated with diminished self-evaluation and feelings of incompetence [3,4].

Analyses of international evidence indicate that healthcare professionals are among the most vulnerable groups to occupational stress and burnout due to high responsibility, continuous patient interactions, emotional workload, and organizational demands [5,6,7,8]. The evidence highlights burnout as a widespread and systemic issue across clinical and public health roles, with significant implications for worker well-being and the quality and safety of health services [9,10].

Community pharmacies in Europe are the most accessible healthcare setting, and pharmacists are the most accessible frontline healthcare professionals [11]. Their role has expanded—from medication supply and safety to counseling, pharmaceutical care, and public health activities—which becomes particularly evident during crises (e.g., epidemics, medicine shortages) [11]. This expanded scope, combined with a dynamic environment, increasing regulatory and administrative requirements, and the need for advanced communication skills, elevates the risk of burnout [12,13].

In Bulgaria, activities in community pharmacies are carried out by two professional groups whose responsibilities stem from their educational qualifications: master pharmacists (MPs), who hold a Master of Pharmacy (MPharm) degree obtained in Bulgaria, and assistant pharmacists (APs), who hold a Bachelor’s degree [14,15,16]. MPs share responsibility for therapeutic outcomes, counsel patients and other professionals, ensure rational and safe medicines use, organize procurement/storage, and carry out prescription and reporting activities [17]. Assistant pharmacists support pharmacists by promoting healthy lifestyle practices, providing professional guidance on the appropriate use and storage of over-the-counter (OTC) medicinal products, dietary supplements, herbal products, medical devices, and cosmetics, and by participating in extemporaneous compounding and logistical activities under the supervision of a master pharmacist [16]. In several countries (e.g., the USA, Germany, North Macedonia), an analogous role is performed by pharmacy technicians, whose support frees pharmacists’ time for specialized activities such as counseling and pharmaceutical care [15,18].

Pharmaceutical care (PC) is the foundation of contemporary practice, with a focus on individualized patient outcomes [11]. High levels of burnout are associated with increased EE and DP and reduced PA, which undermine motivation to provide PC, increase the risk of errors, and impair care quality [3,8,19]. Additionally, chronic stress among personnel is linked to poorer personal health, depressive symptoms, and the risk of addiction [2,20,21].

Although APs do not directly participate in modifying prescribed therapy, they indirectly influence clinical outcomes through shared work environments, support for MPs, and their active role in self-care and health promotion. International research on burnout in community pharmacy practice is less extensive compared with studies among physicians and nurses, and comparisons between MPs and APs remain limited [22]. Existing evidence indicates that administrative burden, frequent interruptions, staff shortages, lack of involvement in decision-making, and deficits in team communication are among the strongest triggers of EE/DP and low PA [23,24,25,26].

In this context, comparative analyses of work characteristics and role-related responsibilities among MPs and APs are needed to identify organizational determinants of burnout, design targeted interventions (workflow structuring, staffing, SOPs, communication standards), and support the sustainable provision of high-quality pharmaceutical care.

The primary objective was to compare burnout levels between master pharmacists and assistant pharmacists working in community pharmacies. The secondary objectives were to assess the impact of work environment characteristics on burnout dimensions and to identify role-specific determinants of occupational burnout.

## 2. Materials and Methods

### 2.1. Study Design

A cross-sectional study was conducted using an anonymous, self-administered online questionnaire, in accordance with the principles of voluntary participation and confidentiality. The study results are reported in accordance with the Strengthening the Reporting of Observational Studies in Epidemiology (STROBE) (University of Bern Institute of Social and Preventive Medicine (Switzerland)) guidelines (Appendix A) [27].

The study included master pharmacists (MPs) and assistant pharmacists (APs) practicing in community pharmacies in the Northeastern region of Bulgaria (the cities of Varna, Ruse, Dobrich, Shumen, Razgrad, Silistra, and Targovishte). The survey link was distributed electronically via email on a single occasion, with the assistance of regional administrative representatives of the relevant professional organizations: the Bulgarian Pharmaceutical Union (representing master pharmacists) and the Bulgarian Association of Assistant Pharmacists (representing assistant pharmacists).

The sampling frame consisted of all master pharmacists (MPs) and assistant pharmacists (APs) registered with their respective professional organizations and actively practicing in community pharmacies in the Northeastern region of Bulgaria (Varna, Ruse, Dobrich, Shumen, Razgrad, Silistra, and Targovishte). At the time of the study, the total number of pharmacists working in community pharmacies in the region was N = 1652, according to official registry data.

The study was carried out between November 2023 and December 2024, after which the survey was closed and the data were statistically processed.

Before the start of data collection, written declarations of institutional consent were obtained at the national level from the presidents of the Bulgarian Pharmaceutical Union and the Bulgarian Association of Assistant Pharmacists. All respondents were informed about the aims, procedures, and intended use of the research results. Prior to participation, each individual provided informed consent. The electronic questionnaire (Google Form) included an information sheet and a mandatory consent form. Participants were required to confirm that they had read the information and agreed to participate voluntarily. Each participant had the right to withdraw at any time without consequences.

No personal identifiable data were collected; a coding system was used to ensure complete anonymity and confidentiality. Only the principal investigator had access to the dataset.

### 2.2. Instrument Description

Data were collected through a structured questionnaire developed by the authors for the purposes of the study. The questionnaire comprised three components: demographic characteristics, burnout assessment using the validated Maslach Burnout Inventory—Human Services Survey for Medical Personnel, and work characteristics assessed using items adapted from internationally recognized European Commission guidelines.

1. Demographic characteristics—gender, age, years of practice, place of practice, and education.
2. Burnout assessment—the validated Maslach Burnout Inventory—Human Services Survey for Medical Personnel (MBI-HSS [MP]) [2] was used to measure three components of burnout: EE, DP, and PA.

The questionnaire contains 22 items rated on a 7-point frequency scale (0—never; 1—a few times a year or less; 2—once a month; 3—a few times a month; 4—once a week; 5—a few times a week; 6—every day).

High scores on EE (≥27) and DP (≥10) and low scores on PA (≤33) are indicative of burnout.

3. Work characteristics—a set of 15 statements, adapted directly from the internationally recognized European Commission guideline “Occupational health and safety risks in the healthcare sector”, was used to assess occupational factors contributing to work-related stress (e.g., physical workload, staff shortages, communication climate). Respondents indicated the extent to which each statement reflected their experience [28].

Completion of the questionnaire required approximately 10–15 min and did not require prior preparation.

### 2.3. Sampling Procedure

Sampling was based on official registry data for master and assistant pharmacists practicing in community pharmacies in Northeastern Bulgaria (Varna, Ruse, Dobrich, Shumen, Razgrad, Silistra, and Targovishte). At the time of the study, the total number of pharmacists working in community pharmacies in the region was N = 1652.

The minimum representative sample size was calculated using the classical formula for finite populations [29], at a 95% confidence interval and ±5% margin of error. The required sample size was N = 312, with an expected proportional distribution of 63% master pharmacists and 37% assistant pharmacists.

A total of 372 respondents participated—221 MPs and 151 APs. The achieved sample exceeds the minimum requirement and can be considered statistically representative, ensuring the reliability of conclusions and generalisations.

### 2.4. Inclusion Criteria

- Practicing in the Northeastern region of Bulgaria (Varna, Ruse, Dobrich, Shumen, Razgrad, Silistra, and Targovishte);
- Full-time employment;
- More than 6 months of professional experience;
- Working in community pharmacies;
- Membership in the relevant professional organization (Bulgarian Pharmaceutical Union or Bulgarian Association of Assistant Pharmacists);
- Provided informed consent.

### 2.5. Exclusion Criteria

- Professionals working outside community pharmacy settings;
- Students, interns, or retirees;
- Specialists with prolonged absence from work (more than three months) due to illness, maternity leave, or other reasons;
- Refusal to participate;
- Incomplete questionnaires.

### 2.6. Data Analysis

Data were processed using IBM SPSS, v.25. Descriptive statistics were applied: quantitative variables are presented as mean (M) and standard deviation (±SD), and categorical variables as frequency and percentage (N, %).

Parametric and non-parametric tests were used to verify hypotheses at a significance level of *p* < 0.05.

To examine the relationship between age, years of practice, and burnout levels in MPs and APs, several statistical models were developed. One-way ANOVA was conducted with age and years of practice as independent variables and EE, DP, and PA as dependent variables.

To analyze the influence of work characteristics on EE, DP, and PA, Levene’s test for equality of variances and the *t*-test for equality of means were applied.

### 2.7. Ethical Considerations

The study was conducted in accordance with the Declaration of Helsinki and was approved by the Research Ethics Committee of the Medical University—Varna, Protocol No. 135/28.09.2023.

## 3. Results

**Figure 1:**
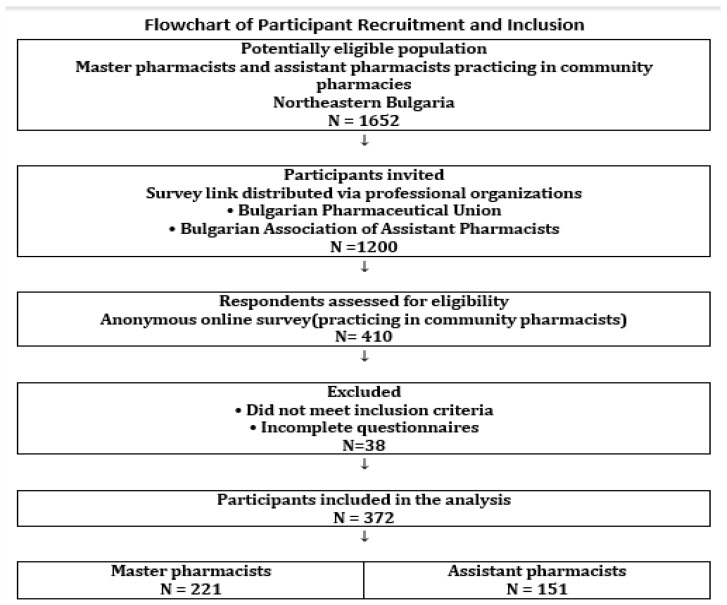
Participant flow diagram.

A total of 372 respondents participated in the study—221 master pharmacists and 151 assistant pharmacists. How the sample size was determined is presented in Figure 1.

The demographic characteristics of the sample are presented in Table 1.

The pharmaceutical workforce was predominantly female. The proportion of males was higher among master pharmacists (23%) compared to assistant pharmacists (13%). The mean age of participants in both groups was ~33–36 years, while the mean years of practice were ~11 for master pharmacists and ~12 for assistant pharmacists.

Among master pharmacists, high levels of emotional exhaustion (EE) were reported by 142 participants, representing 64% of the group. Among assistant pharmacists, 103 participants (68%) reported high EE. High depersonalization (DP) was observed in 117 master pharmacists (53%) and in 85 assistant pharmacists (56%).

The mean scores on the three burnout dimensions (EE, DP, and PA) for both groups are presented in Table 2.

No statistically significant differences were observed between the two groups on any of the three burnout scales: EE (*p* = 0.059), DP (*p* = 0.370), and PA (*p* = 0.771).

Analysis of gender revealed no statistically significant associations with any of the burnout dimensions. Among master pharmacists, the *p*-values were DP = 0.541, EE = 0.001, and PA = 0.135, while among assistant pharmacists, they were DP = 0.48, EE = 0.63, and PA = 0.71. One-way ANOVA analyses examining the effect of age on emotional exhaustion (EE), depersonalization (DP), and personal accomplishment (PA) also revealed no significant associations overall. Among master pharmacists, DP (*p* = 0.941), EE (*p* = 0.366), and PA (*p* = 0.235) were not significantly related to age; among assistant pharmacists, the corresponding *p*-values were DP = 0.310, EE = 0.729, and PA = 0.472. Across both groups, higher burnout scores were most frequently observed in the 20–40-year age group. Furthermore, no statistically significant relationships were found between years of professional practice and any of the burnout dimensions for master pharmacists (DP, *p* = 0.674; EE, *p* = 0.774; PA, *p* = 0.480) or assistant pharmacists (DP, *p* = 0.528; EE, *p* = 0.677; PA, *p* = 0.541).

Nevertheless, in both groups, the highest burnout scores were observed among respondents with up to 10 years of professional experience.

A comparative analysis of the influence of work characteristics on the three burnout scales among master and assistant pharmacists is presented in Table 3.

Among the work characteristics examined, lack of social and communication skills within the team was most strongly associated with burnout across both professional groups. In master pharmacists, it was significantly related to emotional exhaustion (EE, *p* = 0.003) and depersonalization (DP, *p* = 0.001), whereas personal accomplishment (PA) was not significantly affected (*p* = 0.542). In assistant pharmacists, this factor was associated with all three burnout dimensions (EE, *p* < 0.001; DP, *p* = 0.005; PA, *p* = 0.015).

Other characteristics affecting both groups included decision-making without sufficient information on innovations and exclusion from planning and decision-making processes, both of which were linked to elevated EE and DP (MPs: EE, *p* = 0.014 and 0.003; DP, *p* = 0.019 and 0.008; APs: EE, *p* < 0.001 and 0.002; DP, *p* = 0.016 and 0.007, respectively). A negative workplace climate also contributed to higher EE and DP in master pharmacists (*p* < 0.001) and to EE in assistant pharmacists (*p* = 0.002).

Certain work factors were group-specific. Adverse effects of work environment factors influenced EE and DP in master pharmacists (EE, *p* = 0.002; DP, *p* = 0.003) and all three burnout dimensions in assistant pharmacists (EE, *p* < 0.001; DP, *p* = 0.001; PA, *p* = 0.025). Frequent interruptions that hinder efficient and accurate task completion were associated with EE and DP in master pharmacists (*p* < 0.001 and *p* = 0.001, respectively) and with EE and DP in assistant pharmacists (*p* < 0.001 and *p* = 0.018). Conflicting demands were also linked to EE in both groups (MPs, *p* = 0.023; APs, *p* = 0.003).

Several characteristics affected only one professional group. Among master pharmacists, lack of support from colleagues and supervisors and lack of recognition for professional work were associated with both EE and DP (*p*-values 0.001–0.004). In assistant pharmacists, burnout was influenced by strict job requirements (EE, *p* = 0.004; DP, *p* = 0.015), discussion of errors during work (EE, *p* = 0.021), insufficient staffing (EE, *p* = 0.008), physical workload (EE, *p* = 0.009), perceived high responsibility (DP, *p* = 0.043), and time shortages or unrealistic deadlines (DP, *p* = 0.046).

## 4. Discussion

The present study confirms that the workforce in Bulgarian community pharmacies is predominantly female, in line with national statistics and professional registries (~80% women, 20% men) [30].

A substantial body of international research provides robust evidence of high levels of professional burnout among healthcare professionals, particularly physicians and nurses.

A landmark systematic review published in JAMA by Rotenstein et al. demonstrated that the prevalence of burnout among physicians varies widely, ranging from 0% to 80.5%, with most contemporary studies reporting rates exceeding 40%. The authors highlight significant levels of emotional exhaustion, depersonalization, and reduced personal accomplishment across medical specialties, particularly in hospital-based and high-intensity clinical settings [31]. Burnout prevalence of approximately 59.5% among nurses, with high rates of emotional exhaustion (36.1%) and depersonalization (32.4%) [32].

Collectively, these findings demonstrate that professional burnout among healthcare workers is not an isolated phenomenon but a pervasive and escalating public health issue, with serious implications for workforce sustainability, patient safety, and healthcare quality [33,34].

High levels of EE were observed in both master (64%) and assistant pharmacists (68%), with similar DP scores (MPs 53%, APs 56%), consistent with literature indicating that burnout in community pharmacists is primarily driven by emotional exhaustion (68.9%), followed by depersonalization (50.4%) [35,36]. High levels of occupational burnout lead to deterioration in pharmacists’ health and have a negative impact on patients’ health outcomes [37].

The mean burnout scores were similar between master pharmacists (MPs) and assistant pharmacists (APs) across all measured dimensions. For emotional exhaustion (EE), the mean score was 31.79 for MPs and 31.65 for APs. Depersonalization (DP) averaged 13.00 for MPs and 13.33 for APs, while personal accomplishment (PA) scores were 27.90 for MPs and 26.49 for APs. No statistically significant differences were observed between the two groups (*p* > 0.05), suggesting that educational level and professional position within the pharmacy have a smaller impact on burnout intensity than organizational and work environment factors.

Although international studies identify younger age as a risk factor for burnout, with 79% of early-career pharmacists reporting high EE and DP [5,38], this study found no statistically significant relationship between age and burnout. Nevertheless, the highest scores occurred in the 20–40-year age group [39,40,41].

Similarly, no correlation was observed between years of practice and burnout, although higher scores were seen among participants with ≤10 years of experience, consistent with other studies [35,42].

Despite similar levels of burnout among MPs and APs, the work environment factors associated with emotional exhaustion (EE), depersonalization (DP), and reduced personal accomplishment (PA) differ, with workplace stress emerging as the most influential factor in both groups, underscoring the importance of interpersonal relationships and team support in mitigating occupational stress [6,12,43].

Other common factors contributing to burnout included decision-making without adequate information, conflicting or unrealistic demands, exclusion from planning and decision-making, and an adverse work atmosphere.

These findings align with European research linking lack of autonomy and organizational uncertainty to higher EE and DP levels [44].

Burnout among master pharmacists is strongly influenced by a lack of support from colleagues and supervisors (EE *p* = 0.003; DP *p* = 0.004) and insufficient recognition for professional contributions (EE *p* = 0.002; DP *p* = 0.001). Their high level of professional autonomy and responsibility in therapeutic decision-making increases their sensitivity to the organizational climate and the quality of team communication. Additional stressors include specific job demands and the absence of timely information regarding regulatory changes and the implementation of innovations. Similar patterns have been reported internationally; for example, in the U.S., PharmD pharmacists report higher burnout rates (84.7%) than colleagues with bachelor’s or postgraduate degrees (69.1% and 50%), highlighting therapeutic responsibility as a major stressor [40].

In Bulgaria, MPs carry legal and professional responsibility for dispensing medicines, providing counseling, and delivering pharmaceutical care. Lack of recognition and team support can lead to emotional exhaustion and withdrawal from the consultation process, potentially compromising patient care quality [45].

Assistant pharmacists experience a higher physical workload due to staff shortages and routine tasks, such as stock handling, expiration checks, OTC dispensing, dermocosmetics, and preparation of medications [46]. Their limited involvement in decision-making contributes to feelings of powerlessness and reduced professional satisfaction. Key stressors include frequent interruptions, strict requirements, unrealistic deadlines, and perceived excessive responsibility, which can lead to emotional exhaustion and detachment from colleagues and patients. Organizational measures to mitigate these stressors include improving workflow efficiency, clearly defining roles and task responsibilities, providing precise instructions, and standardizing task execution. Additionally, information campaigns and training programs, when implementing new regulations, along with the development of staff communication skills, are recommended to enhance professional competence and reduce burnout risk.

These findings are consistent with international studies showing pharmacy technicians and assistants are exposed to higher physical strain and lower autonomy [47,48]. Although satisfaction with the profession is high (95% of APs report enjoying their work), workload and limited decision-making opportunities increase the risk of emotional and physical exhaustion. Globally, assistant pharmacists are taking on expanded roles, increasing their workload and adding new responsibilities. This has an impact on the level of occupational burnout, particularly on the depersonalization dimension, within the studied sample [49].

Burnout in pharmacists and assistant pharmacists is multifactorial and primarily associated with organizational and communication barriers. Individual factors (age, experience) are less influential, whereas interpersonal relationships and work environment quality are primary determinants. Lack of social support, communication, and recognition for professional contributions are major predictors.

### 4.1. Perspectives for Clinical and Assistive Practice

Enhancing team communication, establishing explicit standards for participation in decision-making, and optimizing workload constitute key preventive measures. The implementation of information campaigns and staff training for new requirements, together with the development of communication skills, is also recommended. Burnout prevention strategies and stress management programs are essential to support the sustainability of pharmacy practice and uphold service quality. Based on the findings of this study, practical prevention strategies will be developed and implemented for pharmacists and assistant pharmacists, and their effectiveness will be evaluated in future empirical research.

### 4.2. Limitations

This study has several limitations that should be considered when interpreting the findings. First, the cross-sectional design precludes any causal interpretation of the observed associations between work-related characteristics and the dimensions of emotional exhaustion, depersonalization, and personal accomplishment. Second, the sample was unevenly distributed across territories, with relatively small representation from smaller towns, which restricted the possibility of conducting robust stratified analyses. In addition, the study was conducted exclusively in Northeastern Bulgaria; therefore, the findings cannot be directly generalised to other regions of the country or pharmacists working in hospital or institutional pharmacy settings without further validation.

Moreover, several potentially influential occupational and personal factors were not measured. Variations in work organisation, such as shift patterns and night work, workload indicators including prescription volume per shift, financial factors such as salary and bonuses, management style, and individual health conditions were not assessed. The omission of these variables may have influenced the observed relationships and should be taken into account when interpreting the results.

The results should be discussed in light of existing literature and the study’s working hypotheses, situating the findings within the broader context of previous research on occupational stress and burnout among healthcare professionals. Particular attention should be given to the practical and theoretical implications of the findings, as well as their relevance for clinical and assistive practice. Finally, future research directions should be highlighted, emphasizing the need for longitudinal and multi-regional studies that incorporate a wider range of organizational and individual factors.

## 5. Conclusions

Although master and assistant pharmacists collaborate closely, differences in professional roles and responsibilities result in varying degrees of impact of specific work characteristics on burnout levels. Among master pharmacists, the primary triggers of high occupational burnout are a lack of support and recognition, as well as insufficient information during the implementation of legislative changes. Among assistant pharmacists, physical workload and the high level of responsibility resulting from insufficient staffing are more pronounced.

Developing strategies for burnout prevention and stress management programs is crucial for the sustainability of pharmacy practice and for improving service quality. Based on this study, practical strategies to prevent burnout among master and assistant pharmacists will be designed and implemented, with their effectiveness evaluated in subsequent empirical research in real-world work settings.

## Figures and Tables

**Table 1:** Demographic characteristics of the sample.

Characteristics		Master Pharmacists		Assistant Pharmacists	
		N	%	N	%
Gender	Female	171	77%	132	87%
	Male	50	23%	19	13%
Age (years)		35.9 ± 0.8		32.5 ± 0.8	
Years of practice		11.08 ± 0.9		11.65 ± 0.7	
Total		221	59%	151	41%

**Table 2:** Comparison of mean burnout scores between master and assistant pharmacists.

Emotional Exhaustion	N	%	M	SD	*p*
Master Pharmacists	221	59	31.79	12.615	0.059
Assistant Pharmacists	151	41	31.65	12.136	
Total	372	100	31.73	12.407	
**Depersonalization**	N	%	M	SD	*p*
Master Pharmacists	221	59	13.00	7.072	0.370
Assistant Pharmacists	151	41	13.33	6.759	
Total	372	100	13.13	6.940	
**Personal Accomplishment**	N	%	M	SD	*p*
Master Pharmacists	221	59	27.90	7.173	0.771
Assistant Pharmacists	151	41	26.49	6.97	
Total	372	100	27.33	7.11	

**Table 3:** Influence of work characteristics on emotional exhaustion, depersonalization, and professional accomplishment.

Professional Role	Master Pharmacists			Assistant Pharmacists		
Scale	EE	DP	PA	EE	DP	PA
M(p)	M(p)	M(p)	M(p)	M(p)	M(p)	M(p)
Physical workload	32.03 (0.859)	13.54 (0.484)	27.85 (0.956)	36.83 (0.009)	14.60 (0.252)	27.60 (0.332)
Insufficient staff	32.81 (0.221)	13.51 (0.271)	28.31 (0.382)	37.38 (0.008)	15.85 (0.37)	28.23 (0.162)
Discussion of errors during work	31.48 (0.062)	12.55 (0.303)	28.15 (0.471)	32.71 (0.021)	13.68 (0.178)	26.69 (0.462)
Lack of time or unrealistic deadlines	32.06 (0.334)	13.08 (0.635)	28.11 (0.192)	33.08 (0.126)	14.36 (0.046)	26.94 (0.404)
Perceived high responsibility	31.86 (0.685)	12.94 (0.533)	27.95 (0.568)	31.9 (0.215)	13.64 (0.0433)	26.68 (0.237)
Strict specific job requirements	31.91 (0.675)	12.92 (0.641)	28.02 (0.47)	32.99 (0.004)	13.97 (0.015)	26.89 (0.144)
Lack of support from colleagues and supervisors	36.09 (0.003)	15.65 (0.004)	27.75 (0.858)	33.97 (0.173)	14.82 (0.118)	27.13 (0.514)
Lack of recognition for professional work	34.37 (0.002)	14.55 (0.001)	28.12 (0.629)	32.25 (0.466)	13.74 (0.535)	27.06 (0.4)
Conflicting demands	33.27 (0.023)	13.68 (0.064)	28.23 (0.368)	35.16 (0.003)	14.23 (0.18)	27.11 (0.366)
Frequent interruptions that adversely affect the efficient and accurate execution of professional duties within the pharmacy setting	33.47 (0.00)	13.81 (0.001)	28.41 (0.062)	37.65 (0.000)	15.07 (0.018)	28.04 (0.41)
Negative workplace climate	40.25 (0.000)	17.21 (0.000)	29.44 (0.092)	38.74 (0.002)	16.26 (0.23)	28.09 (0.234)
Decision-making without sufficient information on innovations	33.41 (0.014)	13.86 (0.019)	28.01 (0.752)	37.58 (0.000)	15.02 (0.016)	27.86 (0.06)
Exclusion from planning and decision-making processes	35.27 (0.003)	14.76 (0.008)	28.09 (0.771)	36.20 (0.002)	15.60 (0.007)	27.42 (0.286)
Adverse effects of work environment factors	34.50 (0.002)	14.48 (0.003)	28.48 (0.245)	38.44 (0.000)	16.22 (0.001)	28.56 (0.025)
Lack of social and communication skills within the team	35.61 (0.003)	15.28 (0.001)	28.34 (0.542)	39.86 (0.000)	16.57 (0.005)	29.36 (0.015)

Note. **EE** = emotional exhaustion; **DP** = depersonalization; **PA** = professional accomplishment; **M** = mean.

## Data Availability

The data presented in this study are available on request from the corresponding author due to privacy and ethical restrictions.

## Abbreviations

The following abbreviations are used in this manuscript:

MBI-HSS (MP)	Maslach Burnout Inventory—Human Services Survey for Medical Personnel
MP	Master Pharmacists
AP	Assistant Pharmacists
EE	Emotional Exhaustion
DP	Depersonalization
PA	Professional Accomplishment

## Appendix A

**Table A1:** STROBE Statement—Checklist of items that should be included in reports of ***cross-sectional studies***.

	Item No	Recommendation	Page No
**Title and abstract**	1	(a) Indicate the study’s design with a commonly used term in the title or the abstract	p. 1
		(b) Provide in the abstract an informative and balanced summary of what was done and what was found	p. 1
**Introduction**			
Background/rationale	2	Explain the scientific background and rationale for the investigation being reported	pp. 2–3
Objectives	3	State specific objectives, including any prespecified hypotheses	p. 3
**Methods**			
Study design	4	Present key elements of study design early in the paper	p. 3
Setting	5	Describe the setting, locations, and relevant dates, including periods of recruitment, exposure, follow-up, and data collection	p. 3
Participants	6	(a) Give the eligibility criteria, and the sources and methods of selection of participants	pp. 3–4
Variables	7	Clearly define all outcomes, exposures, predictors, potential confounders, and effect modifiers. Give diagnostic criteria, if applicable	p. 4
Data sources/measurement	8 *	For each variable of interest, give sources of data and details of methods of assessment (measurement). Describe comparability of assessment methods if there is more than one group	p. 4
Bias	9	Describe any efforts to address potential sources of bias	p. 4
Study size	10	Explain how the study size was arrived at	p. 4
Quantitative variables	11	Explain how quantitative variables were handled in the analyses. If applicable, describe which groupings were chosen and why	p. 4
Statistical methods	12	(a) Describe all statistical methods, including those used to control for confounding	pp. 4–5
		(b) Describe any methods used to examine subgroups and interactions	p. 5
		(c) Explain how missing data were addressed	p. 5
		(d) If applicable, describe analytical methods taking account of sampling strategy	NA
		(e) Describe any sensitivity analyses	NA
**Results**			
Participants	13 *	(a) Report numbers of individuals at each stage of study—e.g., numbers potentially eligible, examined for eligibility, confirmed eligible, included in the study, completing follow-up, and analysed	p. 5
		(b) Give reasons for non-participation at each stage	p. 5
		(c) Consider use of a flow diagram	
Descriptive data	14 *	(a) Give characteristics of study participants (e.g., demographic, clinical, social) and information on exposures and potential confounders	pp. 5–6
		(b) Indicate number of participants with missing data for each variable of interest	p. 5
Outcome data	15 *	Report numbers of outcome events or summary measures	p. 5
Main results	16	(a) Give unadjusted estimates and, if applicable, confounder-adjusted estimates and their precision (e.g., 95% confidence interval). Make clear which confounders were adjusted for and why they were included	p. 6
		(b) Report category boundaries when continuous variables were categorized	p. 7
		(c) If relevant, consider translating estimates of relative risk into absolute risk for a meaningful time period	NA
Other analyses	17	Report other analyses done—e.g., analyses of subgroups and interactions, and sensitivity analyses	NA
**Discussion**			
Key results	18	Summarise key results with reference to study objectives	p. 10
Limitations	19	Discuss limitations of the study, taking into account sources of potential bias or imprecision. Discuss both direction and magnitude of any potential bias	p. 10
Interpretation	20	Give a cautious overall interpretation of results considering objectives, limitations, multiplicity of analyses, results from similar studies, and other relevant evidence	p. 11
Generalisability	21	Discuss the generalisability (external validity) of the study results	p. 11
**Other information**			
Funding	22	Give the source of funding and the role of the funders for the present study and, if applicable, for the original study on which the present article is based	p. 12

* Give information separately for exposed and unexposed groups. Note: An Explanation and Elaboration article discusses each checklist item and gives methodological background and published examples of transparent reporting. The STROBE checklist is best used in conjunction with this article (freely available on the Web sites of PLoS Medicine at http://www.plosmedicine.org/, Annals of Internal Medicine at http://www.annals.org/, and Epidemiology at http://www.epidem.com/). Information on the STROBE Initiative is available at www.strobe-statement.org.
